# The exquisite mechanics of a tsetse bite

**DOI:** 10.7554/eLife.112100

**Published:** 2026-07-02

**Authors:** Katelyn Fealy, Álvaro Acosta-Serrano

**Affiliations:** 1 Department of Biological Sciences, Integrated Biomedical Sciences Graduate Program, University of Notre Dame Notre Dame United States; 2 Department of Biological Sciences, Eck Institute for Global Health, University of Notre Dame Notre Dame United States

**Keywords:** tsetse flies, trypanosomes, blood feeding, feeding apparatus, biting physics, anatomy, Other

## Abstract

Specialized anatomical structures in the mouth and feet of tsetse flies help them feed on blood from a variety of hosts.

**Related research article** Löwe S, Hauf L, Meyer-Natus E, Katiti D, Petersen D, Kovalev A, Büsse S, Steyer A, Matsumura Y, Böhme W, Masiga D, Gorb S, Engstler M. 2025. Anatomy and mechanics of tsetse fly blood feeding. *eLife* **15**:RP110151. doi: 10.7554/eLife.110151.

Tsetse flies are blood-sucking arthropods endemic to sub-Saharan Africa. They are the exclusive carriers or vectors of the parasite *Trypanosoma brucei*, which causes African trypanosomiasis (Wamwiri and Changasi, 2016). This zoonotic, neglected tropical disease can be fatal for humans if left untreated and causes significant economic losses each year due to livestock infections.

Tsetse are obligatory pool feeders, meaning that the fly punctures a host’s skin with its proboscis (a specialised feeding tube) and ingests the blood that pools between cells (Gibson et al., 2017). During feeding, saliva is injected into the skin to prevent blood clotting. If the fly is infected with trypanosomes, the parasites are also injected with the saliva.

Understanding the mechanics of tsetse feeding is particularly important for optimising disease control strategies and for identifying common features shared with other pool-feeding arthropods such as ticks and phlebotomine sand flies. Previous research has thoroughly described the tsetse head structures and feeding behaviours that ensure a successful blood meal (Gibson et al., 2017; Gordon and Crewe, 1948). The fly first uses its mouthparts to probe the host skin surface until it finds a suitable site, where it deeply inserts the proboscis. Subsequently, the fly retracts and protracts the tissue-disrupting proboscis several times. This repeated action causes the tip of the labella (the terminal end of the proboscis) to invert and evert, revealing dorsal rasps and ventral teeth that lacerate blood vessels in the host, generating a larger blood pool (Figure 1). Saliva is delivered simultaneously from the hypopharynx and reingested with the blood meal via a structure known as the cibarial pump.

**Figure 1.**
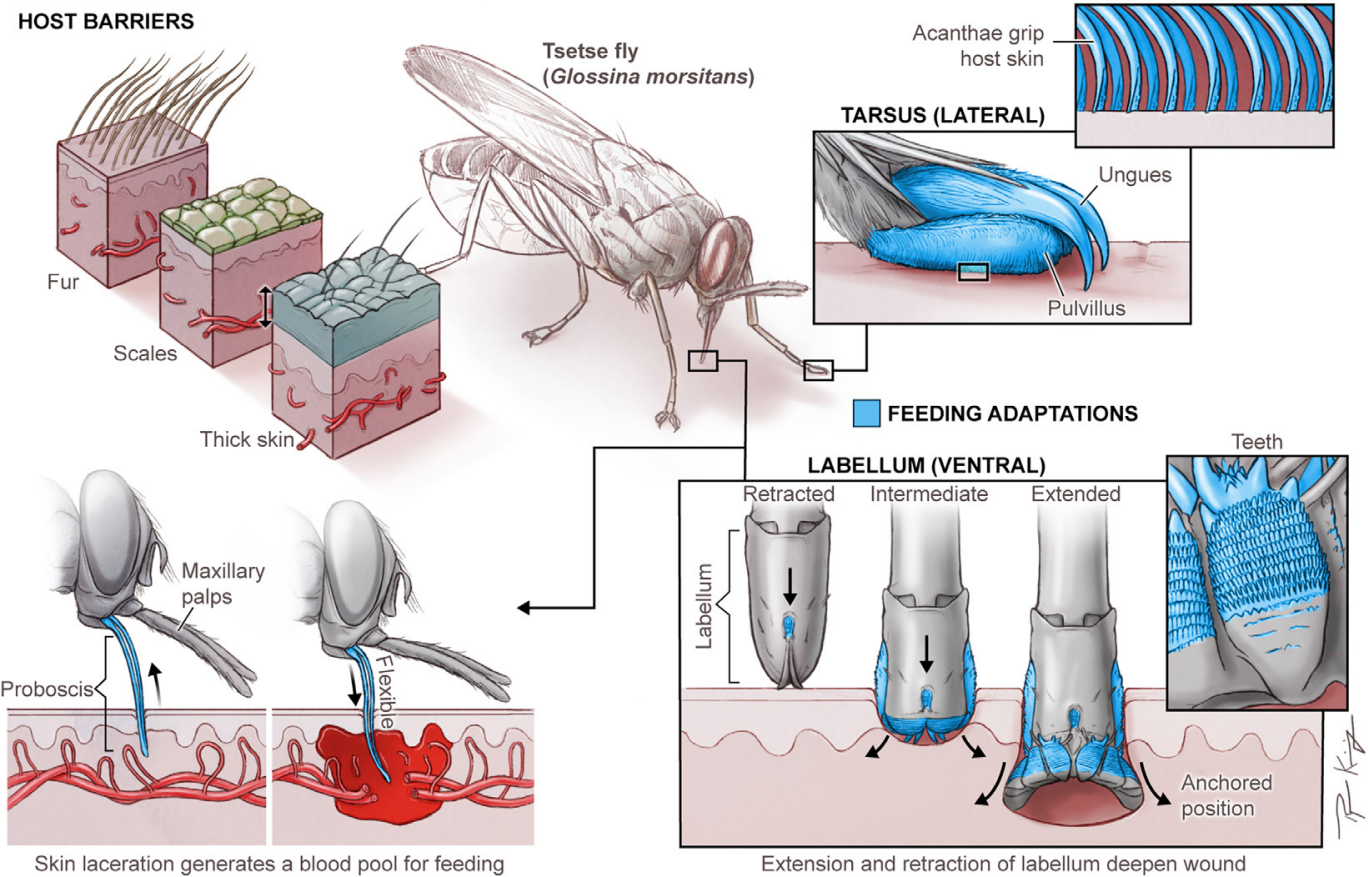
The mechanics of tsetse feeding. *Glossina morsitans* feeds on a wide range of host skin types, including fur-covered, scaled, and thick-skinned surfaces (upper left panel). Successful feeding relies on two sets of coordinated structural adaptations (highlighted in blue). At the tarsus (upper right), paired claws (ungues) grip hair and skin surfaces, while the adhesive pulvillus pad (equipped with thorn-like acanthae) anchors the fly securely to the host during feeding. At the labellum (lower right), a sequence of retraction, intermediate, and extension movements drives the toothed tip of the labellum into the host tissue; once anchored, the teeth lacerate blood vessels to generate a blood pool. The flexible proboscis is then inserted into the wound and partially retracted several times, enlarging the pool and allowing efficient blood uptake via the cibarial pump (lower left). Together, these structures do not operate independently but function as an integrated mechanical system, enabling the tsetse to feed effectively across a remarkable diversity of host skin types.

Image credit: Ryan Kissinger (CC BY 4.0)

These feeding behaviours are severely compromised when a tsetse is infected with trypanosomes. Parasite infection of the salivary glands causes a marked reduction in salivary proteins necessary for feeding, which increases the probing frequency and, in some cases, causes physical damage to the mouthparts themselves (Van Den Abbeele et al., 2010; Gibson et al., 2017; Jenni et al., 1980; Telleria et al., 2014).

In addition to the proboscis and head, the feet (tarsi) of the flies appear to contribute to the feeding behaviour. Prior work has shown that the tarsi appear to provide temperature and neurochemical information necessary for locating and securing a suitable feeding site (Van Haga and Mitchell, 1975; Van der Goes van Naters and Rinkes, 1993). However, little additional work has been done to identify the specific mechanics tsetse use to feed across a wide variety of host skin types, from thin human skin to the tough, scaled integument of reptiles. So how does a tsetse manage to pierce such diverse skin types with apparent ease?

Now, in eLife, Markus Engstler (University of Würzburg) and colleagues – including Stephan Löwe as first author – report new insights on the feeding mechanics of tsetse flies using *Glossina morsitans* as a model species (Löwe et al., 2025).

By combining scanning electron microscopy, confocal imaging, micro-computed tomography and direct biomechanical force measurements, the researchers, who were based at various research institutes in Germany and Kenya, characterised the full feeding sequence from the fly’s landing to blood ingestion. The central finding is both elegant and surprising: rather than relying on any single exceptional morphological innovation, the tsetse achieves remarkable feeding versatility through a suite of subtle, coordinated structural adaptations, including a toothed labellum whose function is more analogous to a chainsaw than a scalpel. Furthermore, the finding that the retractive forces applied to the proboscis consistently exceed the applied penetrative forces suggests that backward pulling is the primary mechanism of wound creation (Figure 1). By moving beyond descriptive anatomy into quantitative functional analysis, the work of Löwe et al. represents a significant advance over prior work (Gibson et al., 2017; Gordon and Crewe, 1948) and establishes a valuable mechanistic framework for studying blood feeding across insect vectors more broadly. No single anatomical structure enables tsetse to feed successfully; rather, multiple physical structures must work in concert to achieve what appears, from the outside, to be a deceptively simple act.

Beyond the mechanics of penetration, the study breaks new ground by quantifying tsetse attachment forces across diverse substrates, demonstrating that grip performance is modest compared with permanently host-attached ectoparasites such as lice (Büscher et al., 2022). This is consistent with the fly’s strategy of brief, opportunistic feeding rather than prolonged host attachment. Capturing trypanosomes within the hypopharynx during active feeding also offers a striking visual window into the moment of parasite inoculation.

An important question for future work will be whether the age of a fly or a trypanosome infection alters these finely tuned feeding mechanics, given that infection is known to affect salivary gland function and feeding duration and mouthpart integrity (Van Den Abbeele et al., 2010; Gibson et al., 2017), depending on the parasite species. Together, these exquisite findings contribute to our understanding of how a blood-feeding fly achieves host versatility without morphological specialization, with broad implications for vector biology and the study of vector-borne pathogens.

**Acknowledgement: **We thank Dr Lee R. Haines and Ms Stephanie Morgan for critical reading of the manuscript, and Mr Ryan Kissinger (http://www.ryankissinger.com/) for the outstanding artwork and valuable input.
